# Submandibular gland preservation versus removal during neck dissection in oral squamous cell carcinoma: a systematic review and meta-analysis

**DOI:** 10.3389/fonc.2026.1818542

**Published:** 2026-06-15

**Authors:** Sargon Shazo, Raji Ranganathan, Giulio Cirignaco, Valentino Vellone, Giulia Romanelli, Pedro Sampaio, Alexandros Damalas, Julio Acero

**Affiliations:** 1 Department of Oral and Maxillofacial Surgery, College of Dentistry, University of Duhok, Duhok, Iraq; 2 Department of Oral Medicine, University College London, London, United Kingdom; 3 Department of Medicine, Section of Maxillo-Facial Surgery, University of Siena, Siena, Italy; 4 Department of Life Science, Health and Health Professions, Università degli Studi “Link”, Rome, Italy; 5 Department of Pediatric Dentistry, Centro Interdipartimentale di Ricerca (C.I.R.) Dental School Lingotto, Universita degli Studi di Torino, Turin, Italy; 6 Faculdade de Odontologia do Recife, Recife, Brazil; 7 School of Dentistry, National and Kapodistrian University of Athens, Athens, Greece; 8 Department of Oral and Maxillofacial Surgery, Ramon y Cajal and Puerta de Hierro University Hospitals, Madrid, Spain; 9 Ramon y Cajal Research Institute (IRYCIS), University of Alcala, Madrid, Spain

**Keywords:** oral squamous cell carcinoma, submandibular gland excision, submandibular gland preservation, survival, xerostomia

## Abstract

**Introduction:**

Submandibular gland (SMG) resection is traditionally included in level IB neck dissection for oral squamous cell carcinoma (OSCC), although isolated SMG metastases are rare. Routine removal may contribute to xerostomia and a reduced quality of life. This systematic review and meta-analysis assessed whether SMG preservation compromises oncologic outcomes or improves functional outcomes.

**Methods:**

This systematic review and meta-analysis followed PRISMA 2020 and Cochrane guidelines, with prospective registration in PROSPERO (CRD420251027851). The literature search included PubMed, Scopus, and the Cochrane Library up to March 2025. Eligible studies compared oncologic or functional outcomes after SMG preservation versus resection in OSCC. Risk of bias was evaluated using the ROBINS-I tool, and evidence certainty was assessed with the Grading of Recommendations Assessment, Development and Evaluation approach.

**Results:**

Eight studies comprising 1,220 patients met the inclusion criteria. Preservation of the SMG did not increase locoregional recurrence (RR 1.05, p = 0.81), affect disease-specific survival (RR 0.86, p = 0.69), or alter overall survival (RR 0.80, p = 0.57) compared with gland resection. Oncologic safety was maintained in early-stage OSCC (T1–T2 N0). Xerostomia outcomes were variably reported across four studies; while some studies suggested a possible trend toward reduced postoperative dry mouth with SMG preservation, findings were inconsistent due to differences in xerostomia assessment methods and patient cohorts.

**Conclusion:**

Based on low-certainty evidence, SMG preservation during neck dissection appears not to compromise oncologic outcomes in selected cases. However, the impact on xerostomia remains inconclusive given the heterogeneity among studies.

**Systematic review registration:**

https://www.crd.york.ac.uk/prospero/display_record.php?ID=CRD420251027851, identifier CRD420251027851.

## 1 Introduction

Oral squamous cell carcinoma (OSCC) is one of the most common head and neck cancer, with approximately 389, 846 new cases and 188, 438 deaths annually worldwide (1). Despite advances in multimodal therapy, the prognosis of OSCC is influenced by different factors, including tumor characteristics, treatment strategies, and host immune status. Regional lymph node metastasis is one of the most significant prognostic factors, reducing survival rates by approximately 50% (2). Accordingly, management of the neck is a critical component of curative treatment for OSCC, with surgery still being the cornerstone of treatment to improve survival and regional disease control (3). The classic radical neck dissection, first described by Crile in 1906 (4), involved en bloc removal of both lymphatic and non-lymphatic structures, providing effective oncologic control but with significant morbidity, most notably shoulder dysfunction due to spinal accessory nerve sacrifice (5, 6).

Modern neck dissections aim to maintain oncologic efficacy while minimizing collateral damage by preserving the spinal accessory nerve, internal jugular vein, sternocleidomastoid muscle, and other non-lymphatic tissues, when appropriate (7, 8). In contemporary practice, level I/IB management of the neck may be performed within different extents of neck dissection (e.g., selective/supraomohyoid dissections, modified radical neck dissection, or more comprehensive dissections), and the submandibular gland is frequently removed during level IB dissection despite being a non-lymphatic structure because of concerns that it could harbor metastatic cancer spread from oral tumors (9). Depending on tumor subsite, laterality, and midline proximity, neck dissection may be performed unilaterally or bilaterally; these aspects are clinically relevant when interpreting both oncologic and functional outcomes in patients with OSCC (10, 11). However, recent evidence has questioned the routine practice concerning the surgical removal of the SMG as a part of the neck dissection Fives et al. found no evidence of intraglandular lymph nodes within the SMG that could serve as a route for metastasis (12, 13). Retrospective studies have also supported the impression that SMG invasion is uncommon in the absence of direct extension from the primary tumor (14–19). In recent years, organ preservation has become one of the most extensively researched and debated topics in the management of head and neck cancer. The submandibular gland (SMG), a major salivary gland, is responsible for 70–90% of the basal salivary flow (20), its preservation during neck dissection has been proposed as a method to minimize postoperative complications, such as xerostomia, dysphagia, and impaired quality of life (21, 22).

Xerostomia is particularly consequential because the SMG provides the majority of resting salivary flow; its removal can lead to persistent dry mouth with significant impacts on speech, swallowing, nutrition, and oral health. Despite these theoretical functional benefits, surgeons have been cautious in adopting SMG-sparing neck dissection because of uncertainty regarding its oncologic safety. The central concern is that leaving the gland *in situ* might increase the risk of locoregional recurrence or residual disease in level IB, which could adversely impact survival (16). To date, the impact of SMG preservation on oncologic outcomes, such as recurrence rates and survival, has not been clearly established, and practice patterns remain variable. To address this issue, we performed a comprehensive systematic review and meta-analysis of the literature, comparing SMG preservation versus SMG resection during neck dissection in patients with OSCC. Through this analysis, we aimed to provide evidence-based guidance on whether preserving the SMG is a safe oncologic practice and whether it confers measurable benefits to the patient’s quality of life.

## 2 Materials and methods

This systematic review with meta-analysis was conducted in accordance with the Cochrane Collaboration and the Preferred Reporting Items for Systematic Reviews and Meta-Analyses (PRISMA) guidelines (23). The protocol was prospectively registered in the PROSPERO database (CRD420251027851). The full protocol can be accessed at: https://www.crd.york.ac.uk/prospero/display_record.php?ID=CRD420251027851.

### 2.1 Eligibility criteria

The Population, Intervention, Comparison, and Outcomes (PICO) framework was applied to guide study selection according to the pre-registered research question: P (Population): Adult patients (≥18 years) with primary oral squamous cell carcinoma (OSCC) undergoing neck dissection that included management of level I/Ib. I (Intervention): Intentional SMG preservation during neck dissection, defined as the gland being deliberately spared and left *in situ* while level Ib nodal and fibrofatty tissue was cleared. C (Comparison): SMG resection (excision) as part of neck dissection. O (Outcomes): Locoregional recurrence rate, regional (neck) recurrence rate, disease-specific survival (DSS), overall survival (OS), and/or xerostomia severity or other validated quality-of-life measures related to salivary gland function.

Inclusion criteria were: (1) randomized controlled trials and non-randomized comparative studies reporting outcomes of SMG preservation versus SMG resection during neck dissection in patients with primary OSCC; (2) studies including adult patients (≥18 years) with histologically confirmed OSCC; (3) studies reporting at least one pre-specified oncological or functional outcome; and (4) publications in English, with no restriction on year of publication.

Studies were excluded if they (1) included patients treated in a salvage setting or with prior head and neck surgery, chemotherapy, or radiotherapy before the index neck dissection (including prior irradiation or neoadjuvant treatments), because these scenarios may substantially confound oncologic and functional outcomes; postoperative adjuvant radiotherapy and/or chemotherapy after the index surgery was allowed and extracted when reported (2) lacked data on any of the specified outcomes of interest; and (3) analyzed duplicate patient populations.

### 2.2 Search strategy and study selection

PubMed, Scopus, and the Cochrane Central Register of Controlled Trials (CENTRAL) were systematically searched from inception to March 2025 for studies published in English, using the following terms: “Squamous Cell Carcinoma of Head and Neck”, “oro-pharyngeal”, oral squamous cell carcinoma”, “Submandibular Gland Excision”, “submandibular gland sparing”, submandibular gland”, “gland-sparing”. A manual search of the reference lists of the included studies was also performed to identify additional relevant studies (backward snowballing). The complete electronic search strategy is presented in **Table 1**. Study selection was independently conducted by two authors (S. S. and R.R.) using predefined inclusion and exclusion criteria. Any disagreements were resolved through discussion and consensus with a third reviewer (G. C.).

**Table 1:** Search strategy.

PubMed	(“Squamous Cell Carcinoma of Head and Neck”[Mesh] OR “Oral cancer” OR “oropharyngeal squamous cell carcinoma” OR “oro-pharyngeal” OR “oral squamous cell carcinoma” OR “head and neck” OR “neck dissection”) AND (submaxillary OR “Submandibular gland preservation” OR “Submandibular Gland Excision” OR “submandibular gland sparing” OR “gland-sparing” OR “submandibular gland”)
Cochrane Central Register of Controlled Trials, and Scopus	(“Squamous Cell Carcinoma of Head and Neck” OR “Oral cancer” OR “oropharyngeal squamous cell carcinoma” OR “oro-pharyngeal” OR “oral squamous cell carcinoma” OR “head and neck” OR “neck dissection”) AND (submaxillary OR “Submandibular gland preservation” OR “Submandibular Gland Excision” OR “submandibular gland sparing” OR “gland-sparing” OR “submandibular gland”)

### 2.3 Data extraction and endpoints

The extracted data included study design and setting, patient population characteristics (sample size, age, sex, tumor site, and stage), details of the intervention and comparison (extent of neck dissection, unilateral or bilateral neck dissection, and SMG preservation technique), use of adjuvant therapy (radiotherapy or chemotherapy), length of follow-up, and outcomes of interest (recurrence rates, survival outcomes, xerostomia or salivary function measures, and any quality-of-life assessments). For time-to-event outcomes of disease-specific survival (DSS), and overall survival (OS), we extracted reported hazard ratios or survival rates when available.

For dichotomous outcomes (recurrence and xerostomia), we extracted event counts as reported by each study at the longest available follow-up. Because xerostomia was assessed heterogeneously (symptom report, questionnaires, or institutional grading), we accepted the study-specific definition and, when graded scales were provided, we dichotomized xerostomia as clinically relevant (moderate–severe) versus absent/mild according to the authors’ thresholds. We recorded the assessment tool and timepoint to support interpretation of between-study heterogeneity and we extracted the number of events and total patients in each group.

### 2.4 Risk of bias and quality assessment

Risk of bias assessment was independently performed by two reviewers (R.R., P.S.) using the ROBINS-I (Risk Of Bias In Non-randomized Studies of Interventions) tool (24), with a third reviewer (S.S.) consulting to resolve any disagreements. Each observational study was judged on domains including bias due to confounding, participant selection, classification of interventions, deviations from the intended intervention, missing data, outcome measurement, and reporting. We categorized the overall risk of bias for each study as low, moderate, or high. Publication bias was assessed using funnel plot analysis for the main outcomes to evaluate the symmetric distribution of studies with similar weights.

The certainty of evidence for each outcome was evaluated using the Grading of Recommendations, Assessment, Development, and Evaluations (GRADE) approach (25). Five domains were examined for possible downgrading: risk of bias, inconsistency, indirectness, imprecision, and publication bias. As all the included studies were non-randomized (observational or prospective cohort studies), the initial certainty of evidence for each outcome was graded as low. Downgrades were made based on methodological inadequacies (e.g., selection bias), heterogeneity (e.g., high I ² values), or imprecise effect estimations with wide confidence intervals.

### 2.5 Subgroup analyses

We pre-specified a subgroup analysis focusing on patients with early stage OSCC (generally T1–T2 N0 cases) to evaluate whether outcomes differ in this lower-risk population, as some authors have suggested that SMG preservation is most appropriate for early-stage oral cancers. Studies that exclusively enrolled patients with early stage OSCC were analyzed separately for locoregional recurrence outcomes and compared with studies that included more advanced cases.

### 2.6 Statistical analysis

Pooled quantitative analyses were performed using Review Manager 5.4 (Cochrane Collaboration, Copenhagen, Denmark). We calculated a pooled risk ratio (RR) with 95% confidence intervals (CIs) for dichotomous outcomes (e.g., recurrence rates, survival at specific time points, and incidence of xerostomia) using the Mantel–Haenszel method, comparing the event risk in the SMG-preservation group to that in the SMG-resection group. All meta-analyses employed a random-effects model (DerSimonian and Laird method) to account for potential inter-study heterogeneity. We considered p < 0.05 as the threshold for statistical significance for the effect estimates. Statistical heterogeneity among studies was evaluated using the chi-square (χ2) test and I^2^ statistics, with I^2^ values of approximately 0–25%, 26–50%, and > 50% indicating low, moderate, and substantial heterogeneity, respectively. However, owing to substantial variability in how xerostomia was defined and measured across studies, we did not pool the xerostomia outcomes in a meta-analysis. Instead, xerostomia data were synthesized qualitatively as a narrative summary.

## 3 Results

### 3.1 Literature search and characteristics

**Figure 1:**
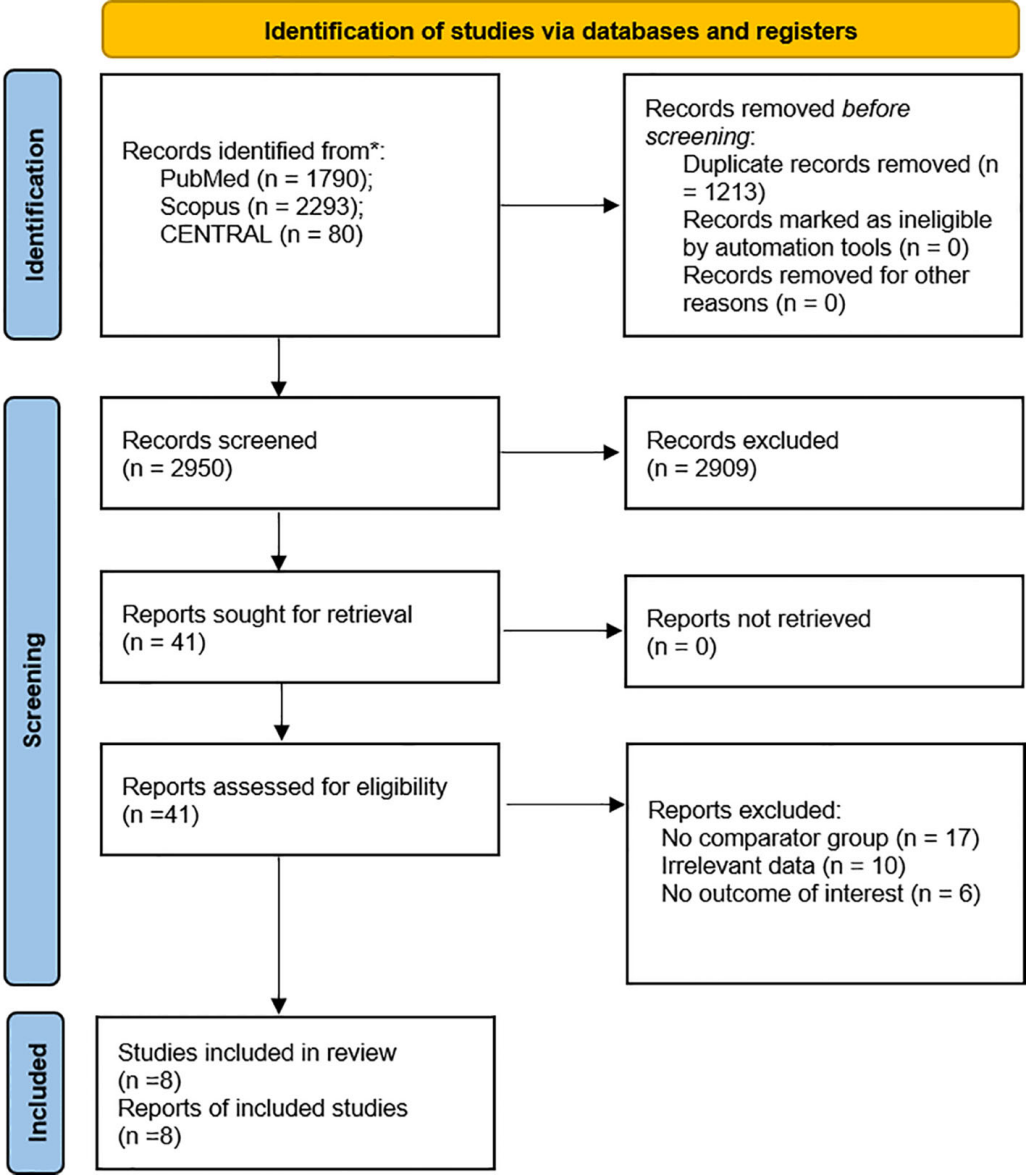
PRISMA flow diagram of study screening and selection.

A comprehensive search of the literature yielded 4163 results. After removal of duplicates (n = 1213) and exclusion by title and/or abstract (2909), 41 studies underwent full-text revision based on our inclusion criteria. Of these, 33 articles were excluded for the following reasons: no comparator group (n = 17), irrelevant data (n = 10), and no outcome of interest (n = 6). Eight studies, including six retrospective studies (26–31) and two prospective cohort studies (32, 33), were eligible for inclusion in the analysis (**Figure 1**), comprising a total of 1,220 patients, of whom 286 (23.4%) had their SMG preserved during neck dissection (SMG preservation group), while 934 underwent standard neck dissection with SMG removal (SMG resection group).

The baseline characteristics of each included study are summarized in **Table 2**. Among the 1,220 included patients, 220 (18.0%) were female and 1,000 (82.0%) were male. The mean age of the patients was approximately 55.83 years in the preservation group and 58.15 years in the resection group. One study included patients with oropharyngeal squamous cell carcinoma (OPSCC) in addition to oral cavity cancers, whereas the others focused exclusively on OSCC. Six of the eight studies specifically analyzed early stage (cT1–T2N0) oral cancers, whereas the remaining studies included patients with a range of tumor stages (including more advanced T3–T4 disease or node-positive cases.

**Table 2:** Baseline characteristics of included studies.

Characteristics	Gu H et al. 2022 (26)		Shih et al. 2023 (31)		Markey et al. 2018 (29)		Gu B et al. 2020 (32)		Lanzer et al. 2014 (30)			Chen et al. 2011 (27)		Du et al. 2020 (28)		Karnati et al. 2024 (33)	
	SMGP (n=36)	SMGR (n=131)	SMGP (n=80)	SMGR (n=74)	SMGP (n=11)	SMGR (n=14)	SMGP (n=31)	SMGR (n=106)	SMGP (n=55)		SMGR (n=113)	SMGP (n=33)	SMGR (n=375)	SMGP (n=29)	SMGR (n=112)	SMGP (n=11)	SMGR (n=9)
**Study design**	Observational		Observational		Observational		Prospective		Observational			Observational		Observational		Prospective	
**Follow-up, months**	12		56.7	56	21	25.7	40.5		44	45		51		35.7		6	6
**Age, years ^§^**	48.6	53.4	56.7	56	67.5	63.3	55.4	60.5	63			50.9	52.11	52.4	64.8	52.15	
**Female, %**	36.1	23.7	5.2	5.4	18	34	35.5	21.7	NR	NR		30.3	13.33	27.6	25.0	20	10
Primary site																	
Tongue	15	51	22	21	11^a^	14^a^	0	0	74^a^			20	188	0	0	11^a^	9^a^
Floor of mouth	5	22	0	0			0	0				0	16	29	112		
Buccal mucosa	9	35	37	33			31	106				8	129	0	0		
Mandible gingiva	7	23	7	12			0	0				2	22	0	0		
Retromolar	0	0	0	0			0	0				0	5	0	0		
Lip	0	0	8	5			0	0				2	5	0	0		
Maxilla	0	0	3	4			0	0				0	0	0	0		
Palate	0	0	3	1			0	0				1	5	0	0		
Clinical Stage																	
I	14	56	49	26	11^b^	14^b^	17	45	26			23	235	22	48	3	5
II	22	75	23	16			14	61	34			10	139	7	64	8	4
III	0	0	3	11	0	0	0	0	33			0	0	0	0	0	0
IV	0	0	4	20	0	0	0	0	86			0	0	0	0	0	0
Grade																	
WD	NR	NR	NR	NR	NR	NR	28	30	NR	NR		28	324	10	39	NR	NR
MD	NR	NR	NR	NR	NR	NR	14	64	NR	NR		4	42	14	59	NR	NR
PD	NR	NR	NR	NR	NR	NR	7	12	NR	NR		1	9	5	14	NR	NR
**Radiotherapy**	0	0	0	0	0	0	4	23	NR	NR		4	39	5	15	2	3

SMGP, submandibular gland preservation; SMGR, submandibular gland removal; NR, Not reported; ^§^ Mean or median; WD, Well differentiated; MD, Moderately differentiated; PD, Poorly differentiated.

^a^Subsite distribution was not reported.

^b^The number of cases in stage I and II was not reported separately.

Across the included studies, gland preservation was performed selectively. Surgeons preserved the SMG only when there were no grossly or intraoperatively suspicious level Ib nodes, no evidence of direct tumour extension into the gland, and when complete periglandular nodal clearance was judged technically feasible. SMG preservation may modestly increase level Ib dissection time and blood loss, but the available comparative data did not show a reduction in lymph node yield. These findings clarify that SMG preservation was intended as organ-sparing level Ib clearance, not as omission of level Ib nodal dissection.

### 3.2 Risk of bias and quality assessment

**Figure 2:**
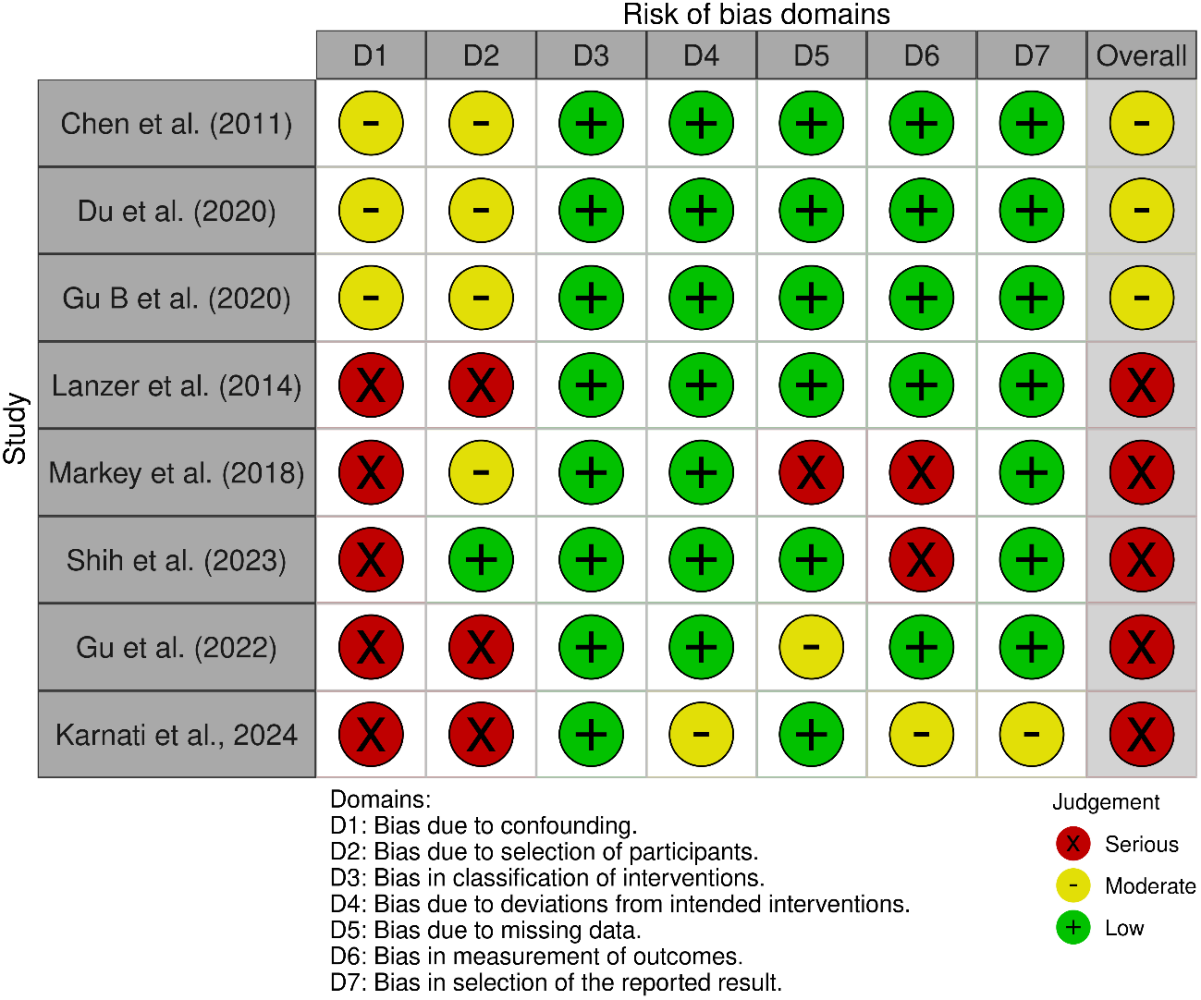
Risk of bias assessment according to ROBINS-I tool.

Using the ROBINS-I tool for observational studies, we found that the overall risk of bias was moderate to high in the included studies. Three studies were judged to have a moderate risk of bias (27, 28, 32) (e.g. they were prospective in design or had better control for confounding) while the remaining five studies had high risk of bias (26, 29–31, 33). Common sources of bias included patient selection bias and confounding. In most retrospective studies, the decision to preserve or remove the SMG was at the surgeon’s discretion, potentially influenced by tumor factors (such as tumor proximity to the gland) or patient factors, which introduces confounding. Blinding of outcome assessment was generally not applicable or not reported, and some studies did not clearly define how outcomes, such as recurrence, were ascertained, raising possible measurement bias. Nonetheless, all included studies reported on our outcomes of interest and were deemed of sufficient quality to contribute to the meta-analysis. A summary of the risk of bias assessment is shown in **Figure 2**.

**Figure 3:**
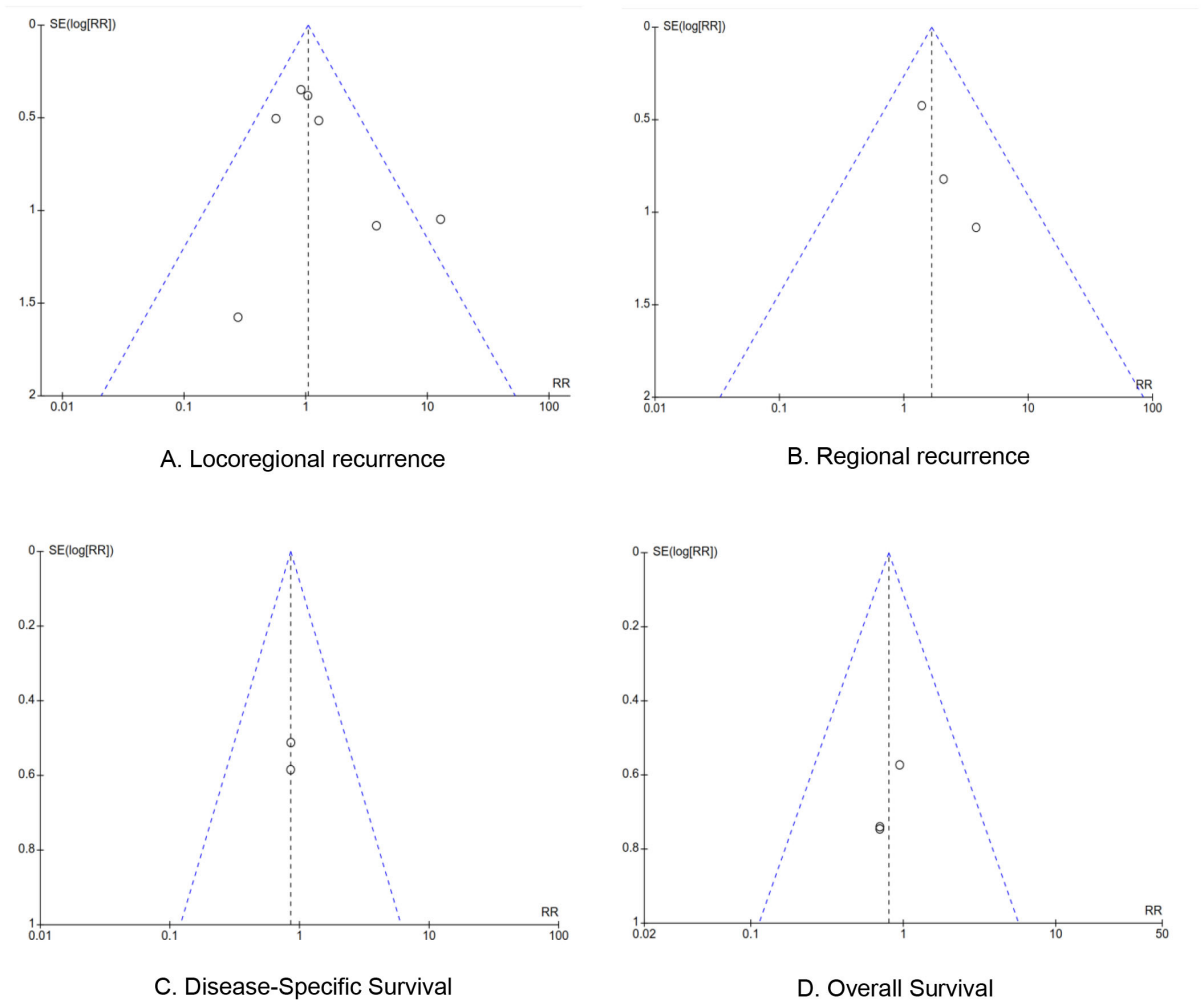
Funnel plots for all outcomes. **(A)** locoregional recurrence, **(B)** regional recurrence, **(C)** disease-specific survival, **(D)** overall survival.

A funnel plot analysis was performed to examine the potential publication bias for survival and recurrence outcomes (**Figures 3A–D**). On visual inspection, the funnel plot for locoregional recurrence appeared asymmetric, with small, imprecise studies (greater SE [log (RR)]) biased predominantly on the right side of the plot, with effect sizes greater than one (RR > 1). In contrast, there was a conspicuous lack of studies with null effects (RR ≈ 1) or reduced risk (RR < 1) in the lower left quadrant, which was composed of small studies. This asymmetry suggests the possibility of publication bias or other small-study effects; that is, studies in which preservation showed no harm may have been less likely to be published. However, given the limited number of studies (less than 10) included in this analysis, these observations are not definitive. We did not perform Egger’s statistical test because of the low number of data points.

The ROBINS-I assessment for methodological quality of the study was used to inform GRADE’s risk of bias domain. The consistent finding of moderate to high risk of bias resulted in a downgrade for risk of bias across all outcomes. Funnel plots were also examined for all outcomes to examine publication bias and level of evidence assessment. Consequently, the certainty of evidence was low across outcomes. The detailed GRADE evidence profile is presented in **Table 3**.

**Table 3:** GRADE Level of evidence assessment.

Outcome	No. of Participants (Studies)	Study Design	Risk of Bias^a^	Inconsistency^b^	Indirectness^c^	Imprecision^d^	Publication Bias^e^	Effect (95% CI)	Certainty
Locoregional recurrence	981 (7 studies)	5 observational & 2 prospective	Serious 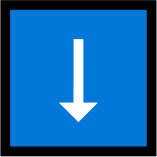	Moderate 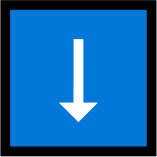	No serious	Serious 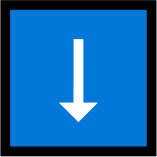	Suspected 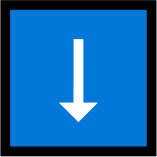	RR = 1.05 (0.71–1.55)	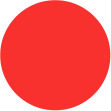 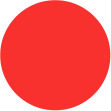 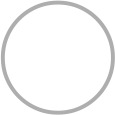 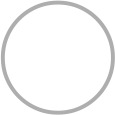 LOW
Regional recurrence	349 (3 studies)	2 observational & 1 prospective	Serious 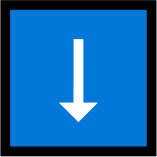	No serious	No serious	Serious 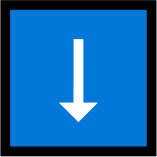	Not detected	RR = 1.68 (0.84–3.37)	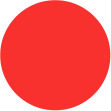 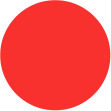 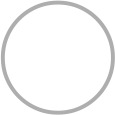 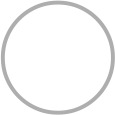 LOW
Disease-Specific Survival (DSS)	278 (2 studies)	1 observational 1 prospective	Serious 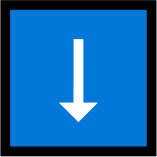	No serious	No serious	Serious	Not detected	RR = 0.86 (0.4–1.82)	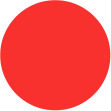 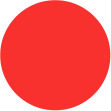 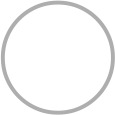 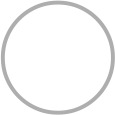 LOW
Overall Survival (OS)	703 (3 studies)	Observational	Serious 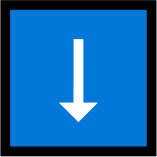	No serious	No serious	Serious 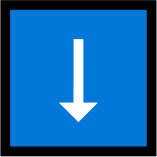	Not detected	RR = 0.8 (0.38–1.72)	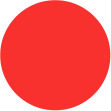 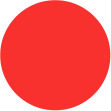 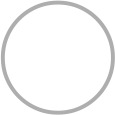 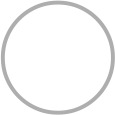 LOW

^a^Downgraded due to moderate to high risk in all included studies as per ROBINS-I (5/8 high risk, 3/8 moderate risk).

^b^Downgraded when heterogeneity was substantial .

^c^No downgrade; population, intervention, and outcomes were directly aligned with the review question.

^d^Downgraded for wide confidence intervals crossing the null.

^e^Downgraded when funnel plots suggested asymmetry, particularly for recurrence outcomes.

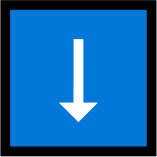
 □ Serious: downgraded for risk of bias due to retrospective design and confounding.

### 3.3 Pooled analysis of all outcomes

#### 3.3.1 Recurrence data

Two studies (30, 31) addressed locoregional recurrence across all stages of OSCC, with 98 patients in the SMG preservation group and 119 patients in the SMG resection group. Locoregional recurrences occurred in 17 and 8 patients, respectively, with no statistically significant difference between the groups (RR 3.39; 95% CI 0.36–31.58; p = 0.28; I² = 74%).

Five studies (27–29, 32, 33) exclusively examined early stage OSCC. In this subgroup analysis, there were 123 patients in the SMG preservation group and 641 patients in the SMG resection group. The incidence of recurrence was 17.1% and 21.5%, respectively, demonstrating no significant difference between the groups (RR 0.9; 95% CI 0.59-1.40, p = 0.65; I² = 0%).

**Figure 4:**
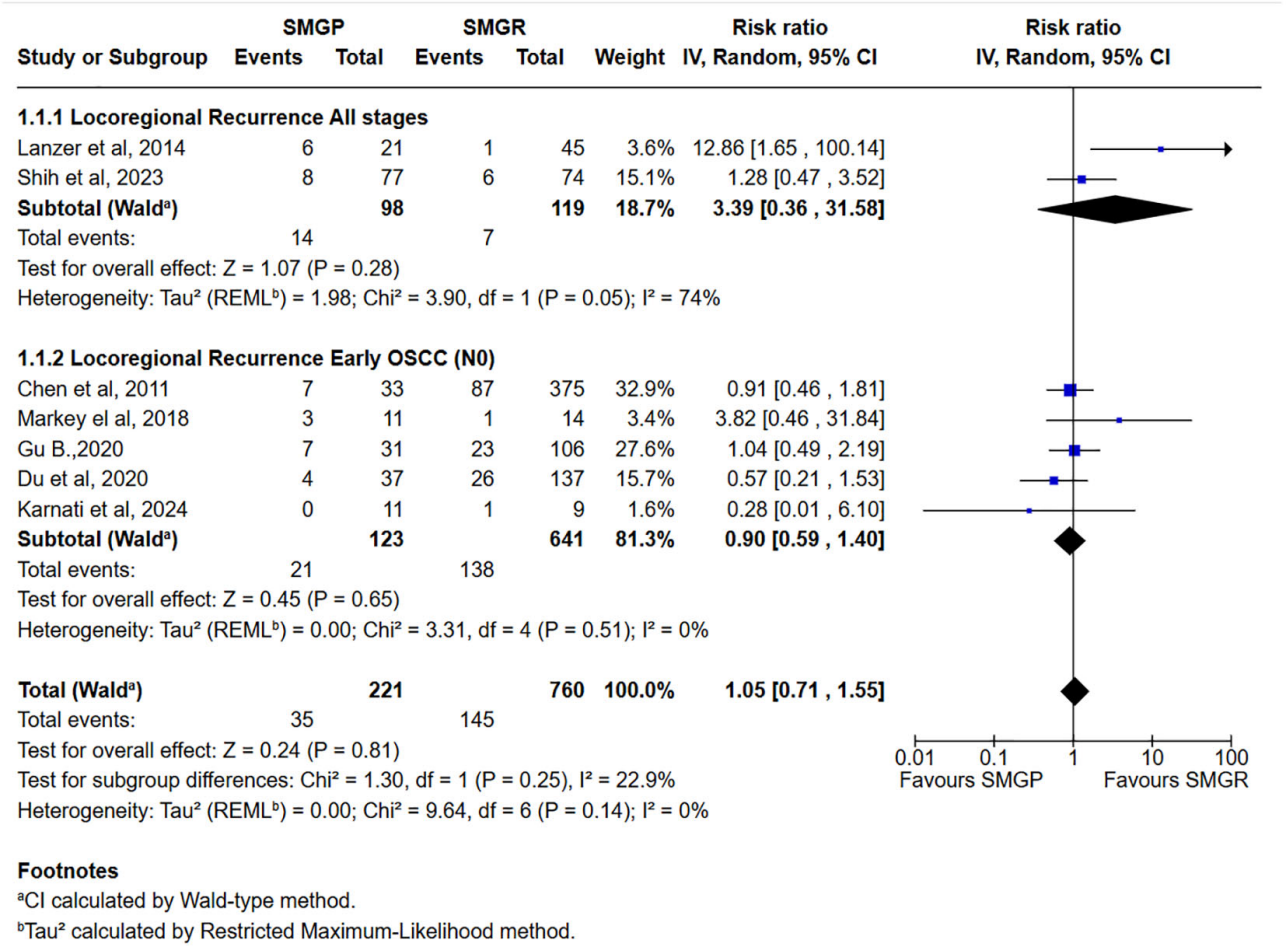
Locoregional recurrence was not significantly different between SMG preservation and SMG resection groups, and the results did not change with subgroup analysis for cases with early OSCC.

The combined analysis of all seven studies showed no significant difference between the two groups (RR 1.05, 95% CI 0.71–1.55, p = 0.81, I² = 0%). The test for subgroup differences also demonstrated no significant difference (Chi² 1.30; df =1, P = 0.25, I² = 22.9). (**Figure 4**).

**Figure 5:**
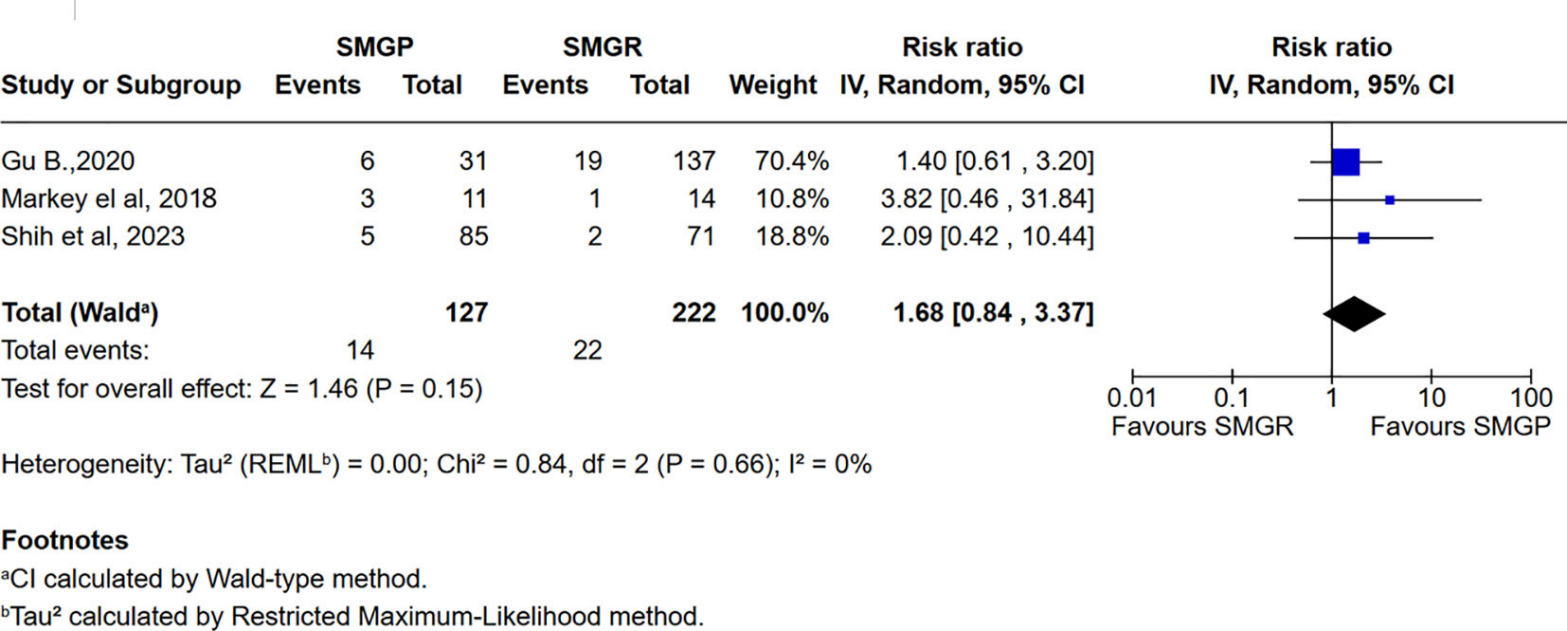
Regional recurrence was not significantly different between SMG preservation and SMG resection groups.

In regard to regional recurrence, three studies reported this outcome separately (29, 31, 32). Similarly, no significant difference was observed between the SMG preservation and resection groups (RR 1.68; 95% CI 0.84–3.37; p = 0.15; I^2^ = 0%; **Figure 5**).

#### 3.3.2 Survival data

**Figure 6:**
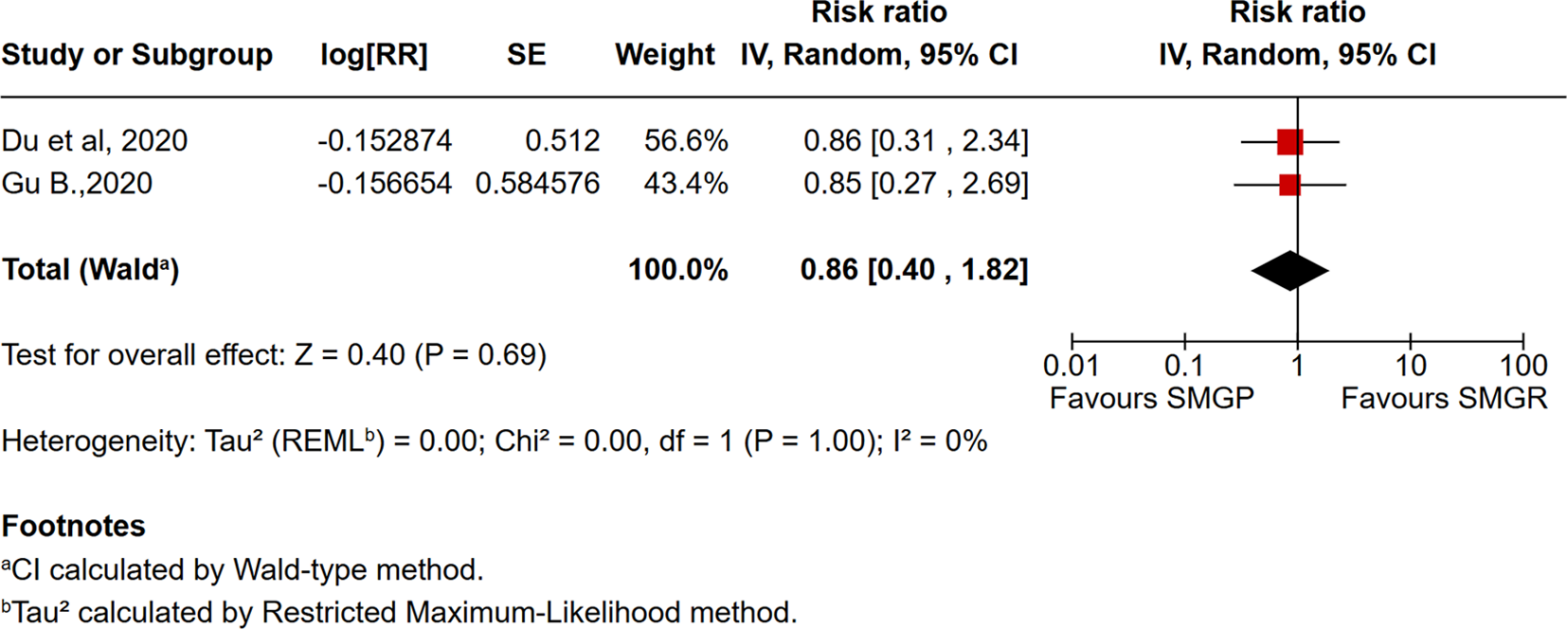
Disease-specific survival (DSS) was not significantly different between SMG preservation and SMG resection groups.

Two studies reported DSS (28, 32), which tracks deaths attributable specifically to cancer. There were 60 patients in the SMG preservation group and 218 in the SMG resection group for the DSS analysis. The pooled rate of cancer-specific death did not differ significantly between the two surgical approaches (RR 0.86; 95% CI 0.40–1.82; p = 0.69; I² = 0%; **Figure 6**).

**Figure 7:**
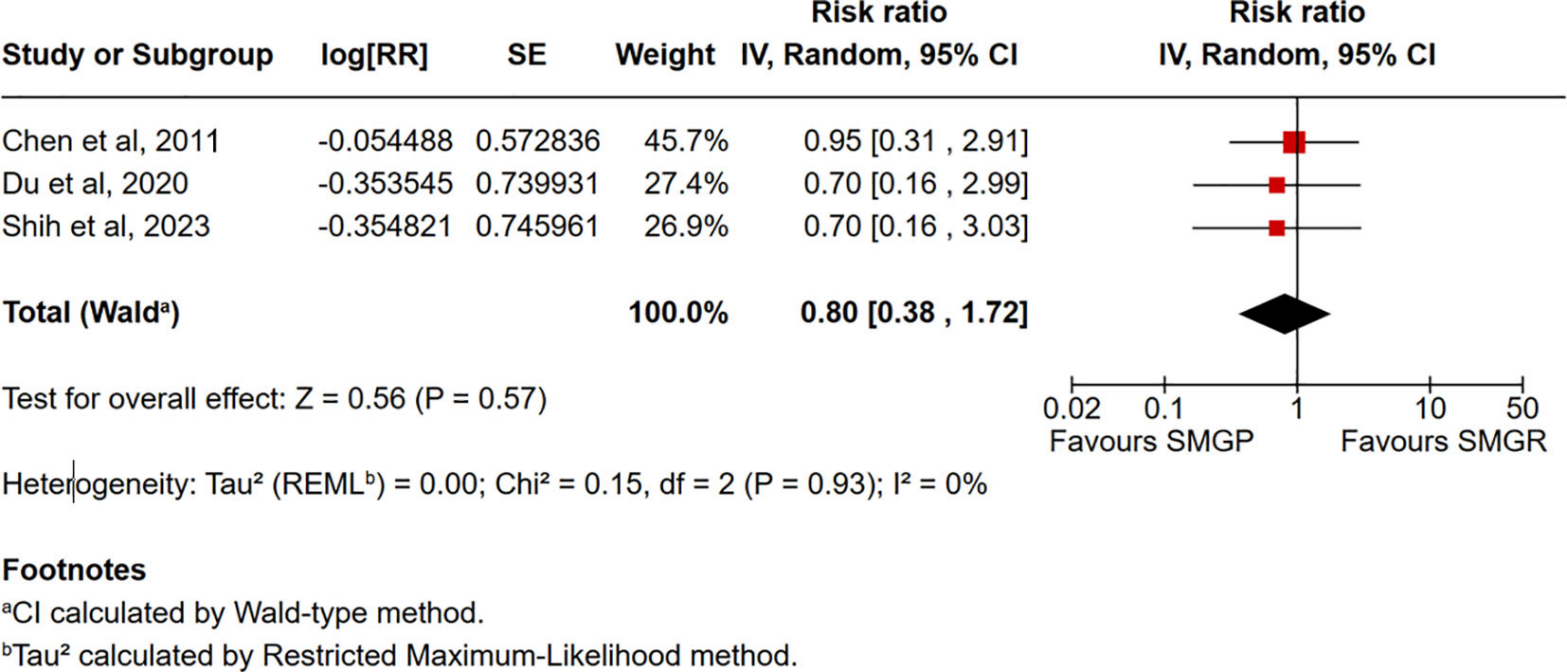
Overall survival (OS) was not significantly different between SMG preservation and SMG resection groups.

Three studies provided data on OS, which accounts for death from any cause (27, 28, 31). Across these studies, the pooled rate of all-cause mortality did not differ significantly between the groups (RR 0.8; 95% CI 0.38–1.72; p = 0.57; I² = 0%; **Figure 7**).

#### 3.3.3 Functional outcomes

Xerostomia was evaluated in four studies (26, 29, 31, 33), although the manner in which salivary dysfunction was assessed varied considerably across cohorts, precluding meaningful quantitative comparison. Differences were noted in outcome definitions, assessment tools, follow-up intervals, and exposure to adjuvant radiotherapy. For these reasons, xerostomia outcomes were examined using a narrative synthesis, the main characteristics and reported xerostomia outcomes of the included studies are summarized in **Table 4**.

**Table 4:** Xerostomia outcomes following submandibular gland preservation versus removal.

Study	Study design/population	Neck dissection (uni/bilateral)	Xerostomia assessment	Radiotherapy status	Key xerostomia findings
Markey et al.	Retrospective cohort; oral/oropharyngeal SCC	Unilateral selective ND only	Patient-reported questionnaire	RT patients excluded	No clear difference
Shih et al.	Retrospective cohort; OSCC	Predominantly unilateral; 8 bilateral ND cases (preservation arm)	Subjective report; saliva expression; scintigraphy	RT patients excluded	Marked reduction in xerostomia with SMG sparing
Gu H et al.	Retrospective comparative study; cT1–2N0 OSCC	Unilateral selective ND	Salivary flow rate; UW-QOL saliva domain	RT patients excluded	Higher salivary flow and QOL with preservation
Karnati et al.	Prospective pilot study; early OSCC (n=20)	Unilateral supraomohyoid ND	Xerostomia Inventory; CODS	RT in minority (not excluded)	Early benefit; no difference at 6 months

Shih et al. (31) investigated postoperative salivary gland function, excluding patient previous radiotherapy, using technetium-99m salivary gland scintigraphy in a subset of patients undergoing submandibular gland preservation. Objective scintigraphy assessment was performed in 36 patients out of 77of the gland preservation cohort, demonstrating preserved gland function in 15 cases and gland hypofunction in the rest of the examined cases; importantly, no cases of severe xerostomia were observed in the preservation group.

Objective salivary measurements were not performed in patients undergoing gland excision, limiting direct functional comparison between treatment arms. Nevertheless, patient-reported xerostomia was assessed in both groups and was more frequently reported following gland excision (37 of 74 patients) than in the preservation group (5 of 71 patients).

Gu H et al. (26) assessed salivary function longitudinally using unstimulated salivary flow measurements obtained in the early postoperative period and during serial follow-up at 3, 6, 9, and 12 months, complemented by patient-reported quality-of-life instruments. Although an early postoperative reduction in salivary output was observed in both groups, patients in whom the submandibular gland was preserved demonstrated recovery of salivary flow during follow-up, whereas reduced salivary output persisted in patients who underwent gland excision. Improvements in saliva-related quality-of-life domains were also reported in the preservation cohort, particularly after exclusion of patients receiving adjuvant radiotherapy.

In contrast, Markey et al. (29) evaluated xerostomia exclusively using patient-reported outcome measures (XeQoL, SF-8, and the Xerostomia Severity Scale), without objective assessment of salivary gland function, no clear difference in subjective dry mouth symptoms was observed between gland preservation and excision groups. Similarly, Karnati et al. (33) assessed xerostomia using a validated xerostomia inventory in a prospective cohort of patients with early-stage (T1–T2 N0) oral squamous cell carcinoma. Xerostomia was evaluated at early and intermediate postoperative time points, early postoperative difference favoring gland preservation was observed, this difference was not sustained at later follow-up.

## 4 Discussion

This meta-analysis systematically evaluated the oncological safety and functional outcomes of SMG preservation versus resection in neck dissection for patients with OSCC. Pooled data from eight observational studies with 1,220 patients revealed no statistically significant differences in locoregional recurrence, regional recurrence, or survival outcomes (DSS, OS) between the SMG preservation and resection groups. An important subgroup analysis of early-stage oral cavity cancers noted a non-significant trend for decreased recurrence with SMG preservation. In functional outcomes, although a functional benefit was expected from gland-sparing surgery, xerostomia outcomes proved difficult to interpret due to substantial heterogeneity in assessment methods, follow-up duration, and radiotherapy exposure. the findings were driven by observational studies with moderate to high risk of bias, limiting confidence in oncologic and functional safety.

The main concern deterring surgeons from routinely preserving the SMG has been the fear of leaving behind cancerous tissue or lymph nodes that could lead to recurrence, and gland removal has been resected as part of an en bloc specimen under the assumption that metastases might involve the gland or adjacent Level Ib nodes nestled against it (10). Despite its reduced morbidity, classical selective neck dissection typically involves the removal of the SMG, a non-lymphatic structure with important physiological roles.

SMG-preserving neck dissection generally involved clearance of the level Ib lympho-fatty tissue while maintaining the submandibular gland capsule and preserving the ductal, vascular, and neural structures required for gland function when technically feasible. In the most detailed operative descriptions, the level Ib packet was dissected from the marginal mandibular nerve, inferior mandibular border, anterior belly of the digastric muscle, mylohyoid muscle, and SMG capsule. Peri-glandular nodal tissue was removed from the superficial plane and, where described, from the tissue deep to the gland by gentle retraction of the SMG. Thus, the nodal tissue between the SMG and the marginal mandibular nerve was intended to be cleared rather than left *in situ*.

The extent of level Ib compartment clearance varied across studies. Chen et al. (27) described capsular dissection around the SMG with removal of the preglandular, post-glandular, prevascular, and post-vascular node groups. Gu B et al. (32) divided level Ib into five positional subgroups around the gland, while Du et al. (28) classified level Ib tissue into six anatomical zones and reported no metastatic involvement in the deep subgroup. Lanzer et al. (30) further highlighted the anatomical concern that lymphatic vessels may adhere to the external capsule of the SMG, particularly in tongue and floor-of-mouth tumors. This supports the concept that oncological risk during SMG preservation is related mainly to the completeness of peri-glandular and pericapsular nodal clearance rather than to proven intraglandular disease.

Anatomical studies have shown that true intraglandular lymph nodes in the SMG are exceedingly rare or nonexistent, indicating that the gland itself is not a typical reservoir for metastasis. Clinical series have consistently reported a very low incidence of isolated SMG involvement by oral cancer, except in cases of direct tumor extension into the gland (15, 17, 34–37). However, Cakir et al. found that the close proximity of the tumor can be a predictor of gland involvement by direct invasion (37). Our meta-analysis reinforces these observations by demonstrating that leaving the gland intact does not lead to higher recurrence rates. In both the overall cohort and early-stage subset, the differences in locoregional control between preservation and removal were not statistically significant.

Reporting of level-specific regional recurrence sites was heterogeneous across the included studies reporting this outcome; however, where data were available, no study documented an isolated SMG-parenchymal recurrence after gland preservation. Chen et al. (27) reported three regional recurrences at ipsilateral levels II, Ib, and Ia, while histopathological examination confirmed no intraglandular metastatic involvement. Gu B et al. (32) reported regional recurrences distributed across levels I-III, with no recurrence arising from within the gland on level Ib subgroup analysis. Du et al. (28) reported no SMG or deep level Ib involvement, and Markey et al. (29) found no regional recurrence in level Ib. Lanzer et al. (30) attributed the higher nodal recurrence risk in tongue and floor-of-mouth cancers to residual pericapsular lymphatic tissue adjacent to the gland rather than to intraglandular disease. In Karnati et al. (33), the reported recurrence in the preservation group involved the submental and sublingual spaces rather than the SMG parenchyma. Overall, the available evidence suggests that the relevant oncological concern is adequacy of peri-glandular nodal clearance and tumor proximity to the gland, rather than documented intraparenchymal nodal disease within the SMG.

Lymph node yield (LNY) is also considered an important prognostic factor. Li et al. concluded that retrieving at least 18 lymph nodes from unilateral neck dissection is the minimum optimal threshold for a better prognosis (38). The LNY is one of the arguments raised by the opponents of SMG preservation. However, a clinical trial by Vetrivel et al. found no significant difference in LNY when comparing two neck dissection techniques for patient with early OSCC, one of which the gland was removed separately (39). Robotic surgery has also been examined as a minimally invasive method to preserve the SMG, and no recurrences were observed during the follow-up period (40).

The available studies reported non-xerostomia morbidity inconsistently, which prevented pooled analysis of these outcomes. The most relevant level Ib complication is marginal mandibular nerve weakness. Gu B et al. (32) reported transient marginal mandibular nerve neuropraxia in 6.5% of the preservation group and 9.4% of the excision group (p = 0.734). Markey et al. (29) observed mild permanent depressor anguli oris weakness in one patient per group and two postoperative seromas distributed equally between groups. Karnati et al. (33) reported longer level Ib dissection time and significantly greater blood loss in the preservation group, but lymph node yield did not differ significantly. Other potential morbidities of neck dissection, including shoulder dysfunction, chyle leak, wound dehiscence, cervical lymphoedema, salivary fistula or sialocele, and contour deformity, were not systematically captured across the included studies. Their comparative impact after SMG preservation versus resection therefore remains uncertain and should be assessed prospectively.

In our study, there were no significant differences in recurrence or survival metrics between SMG preservation and excision, supporting the growing body of literature questioning the traditional practice of routine SMG excision during neck dissection. Our findings corroborate those of Takes et al. (22), who questioned the necessity of gland removal and supported the oncological safety of preservation in appropriately selected patients. Although no statistically significant difference in recurrence was observed between groups in early-stage disease, further investigation is warranted to investigate the influence of a lower tumor burden and reduced risk of microscopic spread in this subgroup.

The interpretation of these results should account for the potential influence of nodal stage and adjuvant radiotherapy, two variables that were not uniformly controlled across the included studies. Six of the eight studies -Gu H et al. (26), Chen et al. (27), Du et al. (28), Markey et al. (29), Gu B et al. (32), and Karnati et al. (33) restricted inclusion to clinically node-negative disease by design, of these six studies, only five reported recurrence outcomes (27–29, 32, 33) which reduces, but does not eliminate, the possibility of N-stage confounding as occult pathological nodal disease and adverse pathological features may still differ between groups. Among studies that included node-positive cases, Lanzer et al. (30) enrolled the broadest nodal spectrum; however, multivariable analysis in that study identified SMG management as an independent factor associated with lymph node recurrence-free survival in the tongue and floor-of-mouth subgroup. Regarding adjuvant radiotherapy, three studies excluded irradiated patients from the functional or oncological analyses (26, 29, 31), while other studies reported broadly comparable radiotherapy exposure between treatment arms. These observations partially mitigate, but do not eliminate, the concern that nodal burden or adjuvant therapy may have confounded the primary oncological comparisons.

Quality of life is another important aspect taken into consideration when treating patients with HNSCC, and adequate salivary function is essential for chewing, taste, and proper swallowing.

Unstimulated (resting) salivary flow is largely driven by the submandibular glands, whereas the parotid glands contribute predominantly to stimulated salivary output. Importantly, minor salivary glands provide a continuous, low-volume secretion that maintains mucosal hydration and oral comfort; therefore, xerostomia severity is not determined by a single gland source but by the overall balance between major and minor salivary contributions. In this context, preservation of even one submandibular gland—as commonly occurs in unilateral neck dissection with ipsilateral gland sparing—may remain clinically meaningful, because it maintains a substantial component of baseline saliva and may mitigate symptom burden, particularly when additional factors (e.g., adjuvant radiotherapy) further compromise salivary function.

One of the motivations for preserving the SMG is to improve postoperative salivary function and related quality of life. Unfortunately, data on functional outcomes were not uniformly reported across studies, limiting our ability to quantitatively synthesize these outcomes. No two studies employed identical methods to measure salivary gland function or patient quality of life, except for the symptom of xerostomia (dry mouth), which was reported in a subset of studies.

The functional impact of SMG preservation extends beyond dry mouth and includes several interrelated quality-of-life domains. Because the SMG contributes a major proportion of unstimulated salivary flow, its removal may impair bolus formation, mucosal lubrication, swallowing, taste perception, speech comfort, oral hygiene, and protection against dental caries. These changes can affect nutrition, social interaction, and overall postoperative recovery. Among the included studies, Gu H et al. (26) provided the clearest quality-of-life data, using the University of Washington Quality of Life questionnaire at 12 months and reporting higher chewing, swallowing, and saliva-domain scores in the preservation group. Other studies used different patient-reported or objective instruments, including xerostomia questionnaires, the Clinical Oral Dryness Score, and salivary scintigraphy. This heterogeneity explains why functional data could not be pooled and highlights the need for standardized salivary and quality-of-life assessment in future studies.

Xerostomia assessment varied across studies and was based on patient-reported outcomes and objective sialometry or scintigraphy measurements. Radiotherapy exposure was excluded in three studies; this represents a potential confounder influencing xerostomia results. Gu H et al. found that, after excluding patients who underwent radiotherapy, the salivary flow rate was significantly higher in those whose glands were preserved at 3, 6, 9, and 12 months post-surgery compared with patient who had their gland removed (26). The authors also used University of Washington Quality of Life (UWQOL) questionnaire to subjectively asses patient quality of life 12 months after surgery and there was significant difference in the domains of chewing, swallowing, activity, and saliva (p= 0.001, 0.005, 0.044, 0.034 respectively), which are directly related to salivary gland function, the authors also found that the preservation group showed significantly better salivary flow and related QoL scores (26). On the other hand, Shih et al. used radiotracer to objectively assess salivary function in patients having their glands preserved after neck dissection and found that none of the patients with spared gland had negative functional results (31).

On the other hand, the smallest studies did not demonstrate a xerostomia benefit. Markey et al. (29). reported virtually no difference in dry mouth symptoms between preservation and resection groups, contrary to physiological expectations. Karnati et al. (33). similarly found no significant xerostomia difference in their 20-patient pilot trial, albeit with only a one-month follow-up. These disparate findings resulted precluding a reliable pooled estimate for xerostomia. In sum, while it is physiologically plausible and suggested in selected studies that preserving the submandibular gland improves salivary function (especially in the absence of radiotherapy), the current evidence for a xerostomia benefit is inconsistent. Preservation of the gland may thus mitigate the debilitating effects of radiation-induced xerostomia, which impacts quality of life, nutrition, and oral health. Future studies should employ objective measures like salivary flow rates and validated patient-reported outcome tools.

Our study has limitations. First, the majority of included studies are observational with retrospective analyses. This introduces inherent risks of bias, especially selection bias and confounding. We attempted to mitigate this by including only comparative studies and by analyzing subgroups, but residual confounding cannot be eliminated. The overall risk of bias was moderate to high in our included studies, which means our conclusions should be interpreted with some caution. Second, there was heterogeneity in patient populations and treatments across studies. The tumor subsites ranged from tongue to floor-of-mouth to buccal mucosa and other subsites, and stages ranged from early T1N0 to more advanced disease. We did perform subgroup analysis on early-stage OSCC and found consistent results in that subset, but the heterogeneity in the advanced stages subgroup analysis (I² ~74% for recurrence outcomes) suggests differences in study conditions. Unfortunately, data were not sufficient to conduct more granular subgroup analyses. Third, the subjective nature of xerostomia reporting represents a major limitation and restricts the strength of inferences that can be drawn. Patient-reported dryness is influenced not only by salivary output but also by assessment timing, baseline oral health, adjuvant radiotherapy, and the extent/laterality of neck dissection, which were not uniformly captured across studies. Moreover, heterogeneity in the instruments used (non-validated symptom queries versus different questionnaire domains or grading systems) reduces comparability and likely contributes to the observed between-study variability. Future studies should adopt standardized, validated patient-reported outcome measures alongside objective assessments (e.g., sialometry and/or salivary gland scintigraphy) at predefined postoperative timepoints to enable more reliable pooled analyses. Other important functional outcomes, such as measured salivary flow rate or comprehensive quality-of-life indices, could not be quantitatively analyzed, and future studies should employ objective quantification such as salivary flow or technetium-99m scintigraphy. Fourth, the follow-up duration in many studies was relatively short (often around 2 years on average). OSCC can have late recurrences beyond 2–3 years, and xerostomia can evolve over time (some patients might partially recover salivary function, or conversely, late fibrosis from radiation can worsen saliva production). Thus, longer follow- up data would strengthen confidence that SMG preservation remains safe and beneficial in the long run. Lastly, we observed signs of publication bias. The asymmetry in the funnel plots suggests that negative or null results may be underreported. We attempted to be comprehensive in our search, including multiple databases and gray literature sources, but bias in the literature itself is always a concern.

Finally the lack of adjusted analyses for the key confounders of nodal stage and adjuvant radiotherapy was another limitation. The included studies did not provide sufficiently homogeneous multivariable-adjusted effect estimates controlling for these factors, precluding a confounder-adjusted meta-analysis. Because all included studies were non-randomized, the decision to preserve the SMG may have been influenced by tumor proximity to the gland, nodal burden, surgeon preference, and anticipated adjuvant treatment. This selection process may have favored gland preservation in patients with more favorable disease characteristics. Future prospective studies should incorporate stratification or randomization according to N stage, primary tumor subsite, and planned adjuvant therapy to address these confounders more directly.

## 5 Clinical implications and future directions

Within the above limitations, our findings have practical implications. The finding that SMG preservation is oncologically safe in early-stage OSCC (T1-T2 N0) supports its selective use in this subgroup, where the risk of occult gland invasion is minimal and functional preservation can meaningfully enhance postoperative quality of life provided that gland integrity is ensured intraoperatively. The evidence suggeststhat SMG preservation may not compromise oncologic control in selected patients; it could also contribute to improved postoperative quality of life through a reduction in xerostomia-related symptom burden.

This is especially pertinent for patients who are expected to undergo postoperative radiation therapy, since preserving at least one SMG could mitigate one of radiation’s most debilitating side effects. For patients with more advanced disease SMG preservation did not appear to worsen the oncologic outcomes, however current evidence is not sufficient to support routine preservation. As always, clinical judgment is paramount—if the gland is suspicious for involvement or very close to the primary tumor, or if level I nodes are grossly involved, oncologic principles would dictate removal of the gland. However, in the many cases where the gland is uninvolved, a selective level Ib dissection that spares the gland is a reasonable and now evidence-supported approach.

To build on the current evidence and address the remaining uncertainties, further research is needed. In particular, future studies should aim to:

Conduct prospective trials: Randomized controlled trials (RCTs) would provide the highest level of evidence. An RCT comparing neck dissection with vs. without SMG removal (especially in early- stage OSCC patients) would help confirm oncologic non-inferiority of gland preservation. Efforts like the trial by Vetrivel et al. are a step in this direction, and additional RCTs or prospective multicenter cohort studies would be valuable.

Stratify by tumor subsite and risk factors: Research should evaluate whether outcomes differ for tumors of the floor of mouth versus tongue, etc. It is possible that floor-of-mouth cancers (which lie anatomically closer to the SMG) might have different considerations than tongue cancers. Similarly, evaluating N0 versus N+ necks separately could be informative.

Include robust functional outcome measures: Subsequent studies should systematically measure salivary gland function (for example, through sialometry or salivary scintigraphy) and use validated quality-of-life questionnaires focusing on xerostomia and related issues. Patient-reported outcome measures should be integrated to capture the subjective benefit.

## 6 Conclusions

In conclusion, within the constraints of low-certainty of evidence, preservation of the SMG during neck dissection appears to be oncologically safe in the treatment of OSCC especially in early stages, and does not appear to increase locoregional recurrence or compromising survival. However, the effect on xerostomia is uncertain: although some studies suggest reduced postoperative dry mouth, evidence remains insufficient for firm conclusions. Each case should be evaluated within a multidisciplinary context, considering tumor characteristics and patient factors, to make an informed decision on SMG management.

## Data Availability

The original contributions presented in the study are included in the article/supplementary material. Further inquiries can be directed to the corresponding author.
